# Dr. William Jacob Coppernoll

**Published:** 1916-12

**Authors:** 


**Dr. William Jacob Coppernoll,** Michigan 1887, a laryngologist of Newark, N. Y., died at Laconia, N. Y., Oct. 14.

